# Efficacy and safety of PDE5-Is and α-1 blockers for treating lower ureteric stones or LUTS: a meta-analysis of RCTs

**DOI:** 10.1186/s12894-018-0345-4

**Published:** 2018-05-03

**Authors:** Xifeng Sun, Wei Guan, Haoran Liu, Kun Tang, Libin Yan, Yangjun Zhang, Jin Zeng, Zhiqiang Chen, Hua Xu, Zhangqun Ye

**Affiliations:** 1 Department of Urology, Tongji Hospital, Tongji Medical College, Huazhong University of Science and Technology, Wuhan, 430030 China; 2 Institute of Urology of Hubei Province, Wuhan, 430030 China

**Keywords:** Adrenergic alpha-1 receptor antagonists, Phosphodiesterase 5 inhibitors, Ureteral calculi, Lower urinary tract symptoms, Prostatic hyperplasia

## Abstract

**Background:**

Lower ureteric stones and lower urinary tract symptoms are common in urology.Drug treatment is one of standard therapy,but the efficacy was controversial.Thus we aimed to investigate the efficacy and safety of monotherapy or combination therapy of adrenoceptor1 blockers and phosphodiesterase5 inhibitors for treatment.

**Methods:**

Randomized controlled trials up to November 2016 were retrieved from PubMed, the Cochrane Library, Web of Science and Embase. A total of 17 studies were included. We analyzed data through random or fixed effect models. The heterogeneity between studies was assessed by the I^2^ test statistic.

**Results:**

As for lower ureter stones, our analysis demonstrated tadalafil had a significantly lower incidence of abnormal ejaculation than adrenoceptor1 blockers (2.31 95%CI 0.22to0.84, *P* = 0.01),while combination therapy had a higher expulsion rate (2.49 95%CI 1.44to4.29, *P* = 0.001) and shorter expulsion time (− 1.98 95%CI -3.08to0.88, *P* = 0.0004) than tamsulosin. As for lower urinary tract symptoms, our analysis indicated adrenoceptor1 blockers was more effective than phosphodiesterase5 inhibitors on decreasing International Prostate Symptom Score (1.96 95%CI 0.03to3.89, *P* = 0.05) and Post-Void Residual (9.41 95%CI 1.40to14.41, *P* = 0.02) and phosphodiesterase5 inhibitors showed a greater effect than adrenoceptor1 blockers on improving Erectile Dysfunction (2.23 95%CI 1.24to3.22, *P*<0.0001).Combination therapy had a significantly better effect on International Prostate Symptom Score (1.47 95%CI 1.25to1.69, *P*<0.0001), Maximum flow rate (0.87 95%CI 0.71to1.04, *P*<0.0001), Post-Void Residual (10.74 95%CI 3.53to17.96,*P* = 0.004) and Quality of life (0.59 95%CI 0.22to0.97, *P* = 0.002) but was associated with higher incidences of adverse events (3.40 95%CI 1.82to6.36, *P* = 0.0001) than adrenoceptor1 blockers. Combination therapy had a significantly better effect on International Prostate Symptom Score (4.19 95%CI 3.34to5.04, *P*<0.0001), Maximum flow rate (1.86 95%CI 1.32to2.39, *P*<0.0001), Post-Void Residual (22.58 95%CI 9.13to36.04, *P* = 0.001) and Quality of life (0.68 95%CI 0.37to1.00, *P*<0.0001) without higher incidences of adverse events than PDE5-Is.

**Conclusions:**

In conclusion, this meta-analysis suggested combination therapy had a best efficacy of therapy for lower ureteric stones or lower urinary tract symptoms correlated with benign prostatic hyperplasia than monotherapy. Adrenoceptor1 blockers was more effective than phosphodiesterase5 inhibitors on International Prostate Symptom Score and Post-Void Residual. Both monotherapy and combination therapy were safe.

**Electronic supplementary material:**

The online version of this article (10.1186/s12894-018-0345-4) contains supplementary material, which is available to authorized users.

## Background

Benign prostatic hyperplasia (BPH) is characterized by nonmalignant hyperplasia of prostatic tissue and is caused by proliferation of smooth muscle (SM) and epithelial cell in the transition zone of prostate.BPH is common in aging men and could result in bothersome lower urinary tract symptoms correlated with BPH (LUTS/BPH) which decrease Quality of life (QoL) by interrupting sleep and daily activities [1, 2]. In the US, approximately 75% of males from 60 to 69 years old and 83% of men aged 70 years or older are estimated to have got LUTS/BPH, and the annual direct medical cost due to management is more than $1.1 billion [3].

Each year, about 0.1% of the adult population of the US are admitted to hospital for treating urinary stones, leading to direct medical costs of more than $2 billion per year [4]. Stone incidence varies by race, ethnicity, geographic region and is higher in mountainous areas and deserts located in the southern US and Central European areas [5]. Nowadays, kidney stones are most prevailing from 20 to 40 years old and the incidence of men are 2 to 3 times higher as compared with women, which may due to less calcium and more citrate excreted by women. About 22% of all urinary calculus locate in the ureter, of which about 68% are found in the distal ureter [6].

In the past 20 years, various therapy methods for LUTS/BPH and lower ureteric stones were developed, which included observation, drug treatment and surgical procedures [7–10]. At present, drug treatment has become standard therapy and is widely recommended by clinical guidelines for LUTS/BPH and lower ureteric stones after series randomized controlled trials (RCTs) revealing the obvious effect of adrenoceptor blockers(ABs) [8]. α-1adrenergic receptors play significant roles in the contraction of SMs of the urinary tract and mainly centralize in the distal ureter, and relaxation of these SMs by blocking the receptors will improve LUTS/BPH and cause ureter dilatation contributing to stone expulsion [11].

Recently, the phosphodiesterase5 inhibitors (PDE5-Is) such as tadalafil, have shown up which could relax the SMs of ureter and prostate by working on nitric oxide cyclic-guanine monophosphate (NO/cGMP) signaling pathway [12–14]. Because of this character, tadalafil was approved by FDA in treating LUTS/BPH, erectile dysfunction (ED) and pulmonary arterial hypertension [15] and PDE5-Is were acknowledged in the guidelines published by the Japanese Urological Association (JUA) and the European Association of Urology (EAU). There is Level 1 evidence supporting the efficacy of PDE5-Is for treating LUTS/BPH [16]. By combining drugs acting through different mechanisms, better relaxation of SMs could be achieved [17]. Like ABs, PDE5-Is have an onset of action that occurs within weeks. However, the two classes of drugs are associated with adverse events (AEs) such as headache, dizziness and hypotension leading to the relatively limited clinical application.

Nowadays, a few meta-analyses had compared the effect of PDE5-Is with ABs in the therapy of LUTS/BPH but meta-analyses about lower ureteric stones were rare. In 2012, Gacci et al. [18] performed a meta-analysis of PDE5-Is plus ABs verse ABs for treating LUTS/BPH. They declared that PDE5-Is might be a treatment option with great promise for patients with LUTS/BPH. Then several clinical trials analyzed the effect of PDE5-Is for LUTS/BPH vs ABs. In 2015, XH Wang et al. [19] conducted an meta-analysis to summarize the comparative effect and safety of monotherapy and combination therapy of PDE5-Is and ABs for LUTS/BPH, in which they suggested that PDE5-Is used alone was effective except for Post-Void Residual (PVR) than ABs and was more effective than ABs on increase of International Index Of Erectile Function (IIEF) score while combination therapy had the best effect. Moreover, either monotherapy or combination therapy was safe. However, the literature searches weren’t extensive enough in the two studies.

Recently, researchers focused on investigating the comparative effect and safety of monotherapy and combined use of PDE5-Is and ABs for treating LUTS/BPH and lower ureteric stones. However, the conclusions were still very controversial. Thus, our study aimed to comprehensively compare efficiency and safety of monotherapy with combination therapy of PDE5-Is and ABs for treating LUTS/BPH and lower ureteric stones based on existing RCTs.

## Methods

All methods for this systematic review and meta-analysis are outlined in a prospectively registered protocol available online [20] (PROSPERO identifier CRD42017059295), and reporting follows Preferred Reporting Items for Systematic Reviews and Meta-Analysis (PRISMA) guidelines.

### Search strategy

According to the PRISMA statement [21], we performed an extensive search of a database of PubMed, the Cochrane Library, Web of Science and Embase up to November 2016. The search terms included the following keywords: (“lower urinary tract symptom” OR “LUTS” OR “benign prostatic hyperplasia” OR “BPH” OR “ureter stone” OR “ureteric stone”) AND (“a-adrenoceptor antagonist” OR “a-adrenoceptor inhibitor” OR “α-blocker” OR “alfuzosin” OR “doxazosin” OR “tamsulosin” OR “silodosin” OR “terazosin”) AND (“phosphodiesterase type 5 inhibitor” OR “sildenafil” OR “tadalafil” OR “mirodenafil” OR “avanafil” OR “udenafil” OR “vardenafil” OR “lodenafil”). Furthermore, the references of selected articles and the abstracts presented at related conferences were also checked by hand to identify additional potential studies. The languages were limited to English.

### Inclusion and exclusion criteria

The inclusion criteria for the studies were as follows: (1) human studies; (2) reporting original research;(3) enrolling patients of LUTS/BPH or lower ureteric stone; (4) reporting evaluation indexes of LUTS/BPH such as IPSS, Qmax, PVR, QoL, IIEF before and after treatment; (5) reporting evaluation indexes of stones such as expulsion rate, expulsion time. Additionally, reviews, superficial abstracts, studies with a sample size< 10 were excluded.

### Selection of studies

Two authors (XFS and WG) respectively screened the title, abstract and results, keywords and conclusion of every single study to identify included articles. Any discrepancies were resolved by discussing together. Full texts were screened to further evaluate whether the article had met the inclusion criteria.

### Data extraction

Two authors (XFS and WG) respectively extracted the required data from the included articles through using a designed tabulation based on the Inclusion criteria and a third author verified the data. Data of different aspects were classified into the corresponding column. Based on the Cochrane Handbook, missing or vague information was imputed and was required from the authors of original articles or other relevant articles when necessary.

### Quality assessment

Two investigators independently assessed the quality levels of the included studies according to the Jadad score, which is based on the following aspects: randomized allocation sequence, allocation concealment, blinding and quitting. Studies with scores of 4 points or higher were considered to be of high quality.

### Statistical analysis

All analyses were conducted applying Cochrane Collaboration review manager software (RevMan5.3). The pooled effects were calculated as weighted mean difference (WMD) for continuous variable and odds ratio (OR) for dichotomy variable, as well as 95% confidence intervals (Cls). We chose two-sided in all test and *P* < 0.05 were considered statistical significance. The heterogeneity was determined by the Cochrane’s Q-statistic test [22], and the inconsistency was quantified with the I^2^ statistic. When I^2^ > 50% or P_Q_ ≤ 0.1, which suggested substantial heterogeneity, the random-effects model (DerSimonian-Laird method) was applied [23]; otherwise, the fixed-effects model (Mantel-Haenszel method) was applied [24]. Sensitivity analyses were conducted by sequentially excluding each study to validate the reliability of the results and analyse the heterogeneity. Evaluation of safety was conducted via comparing the AEs, and the indexes could be calculated when at least 2 studies contained relevant data.

## Results

### Search results

**Fig. 1:**
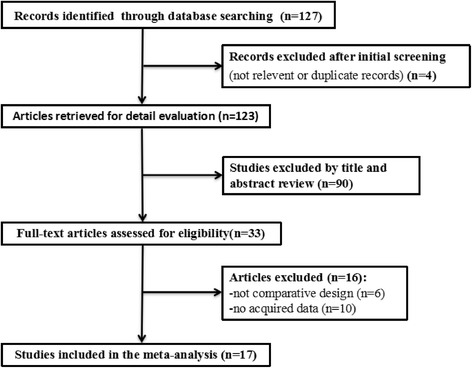
Flow chart of study selection

Figure 1 displayed the study selection process. Of 127 retrieved articles in initial search, 17 RCTs finally met full inclusion criteria via full-text evaluation from 33 potentially eligible articles for this systematic review and meta-analysis.

### Study characteristics and quality assessment

Tables 1 and 2 list the characteristics of the included studies in the meta-analysis. Regarding the lower ureteric stones, 5 studies [6, 13, 25–27] including a total of 861 patients were available. Regarding the LUTS/BPH, 12 studies [28–39] including a total of 1052 patients were available. As for bias, we gave positive appraise for all the selected studies. Additionally, 13 [6, 13, 25–30, 33, 35, 37, 38, 40] included studies were of high quality and 4 [31, 32, 34, 36] were of low quality according to the Jadad scores [41].

**Table 1:** Characteristics of the included studies of lower ureteric stones in meta-analysis

Author	Year	Country	Characteristics of participants		Design	Intervention	No.	Study interval	Comparable index	Jadad score
			location	size						
Kumar	2015	India	distal ureteric stones	5-10 mm	RCT	tamsulosin 0.4 mg qd tadalafil 10 mg qd silodosin 8 mg qd	90 90 90	4 weeks	age,gender,stone size,expulsion rate,expultion time,analgesic use,AEs	6
KC	2016	Nepal	distal ureteric stones	5-10 mm	RCT	tamsulosin 0.4 mg qd tadalafil 10 mg qd	41 44	2 weeks	age,gender,stone size, expulsion rate, expulsion time,analgesic use,AEs	4
Puvvada	2016	India	distal ureteric stones	5-10 mm	RCT	tadalafil 10 mg qd tamsulosin 0.4 mg qd	100,100	4 weeks	age, gender, stone size, expulsion rate, expulsion time, analgesic use, AEs	6
Kumar	2014	India	distal ureteric stones	5-10 mm	RCT	tamsulosin 0.4 mg qd + tadalafil 10 mg qd	31	6 weeks	age,gender,stone size, expulsion rate, expulsion time,analgesic use, AEs	4
						tamsulosin 0.4 mg qd	31			
Jayant	2014	India	distal ureteric stones	5-10 mm	RCT	tamsulosin 0.4 mg qd + tadalafil 10 mg qd	122	4 weeks	age, gender,stone size, expulsion rate, expulsion time, analgesic use, AEs	5
						tamsulosin 0.4 mg qd	122			

*/* not available, *AEs* adverse events, *RCT* randomized controlled trial

**Table 2:** Characteristics of the included studies of LUTS/BPH in meta-analysis

Author	Year	Country	Characteristics of participants					Design	Intervention	No.	Study interval	Comparable index	Jadad score
			Age	Cause of LUTS	IPSS	ED	Sick time						
Abolyosr	2013	Egypt	≥45	BPH	≥7	Yes	≥3 months	RCT	Doxazosin 2 mg qd Sildenafil 50 mg qd Combination	50 50 50	4 months	IPSS, PVR, Qmax, IIEF, QoL	4
Kaplan	2007	USA	50–76	BPH	17.4(mean)	Yes	/	RCT	Alfuzosin 10 mg qd Sildenafil 25 mg qd Combination	20 21 21	12 weeks	IPSS, Qmax, Nocturia, PVR, IIEF, AEs	3
KIM	2011	Korea	≥45	BPH	≥13	/	≥6 months	RCT	Tadalafil 5 mg qd	51 49	12 weeks	IPSS, QoL, Nocturia, Qmax, PVR, AEs	5
									Tamsulosin 0.2 mg qd				
Singh	2014	India	≥45	BPH	> 8	/	≥6 months	RCT	Tamsulosin 0.4 mg qd Tadalafil 10 mg qd Combination	45 44 44	3 months	IPSS, Qmax, PVR, QoL, IIEF, AEs	3
Tuncel	2010	Turkey	47–77	BPH	> 12	Yes	≥6 months	RCT	Sildenafil 25 mg qd 4d/week	20	8 weeks	IPSS, Qmax, PVR, QoL, IIEF	4
									Tamsulosin 0.4 mg qd Combination	20 20			
Bechara	2008	Argentina	> 50	BPH	> 12	/	≥6 months	RCCT	Tamsulosin 0.4 mg qd + tadalafil 20 mg qd	27	45 days	IPSS, Qmax, PVR, QoL, AEs	5
									Tamsulosin 0.4 mg qd + placebo	27			
Liguori	2009	Italy	50–75	BPH	> 8	Yes	≥6 months	MRCT	Alfuzosin 10 mg qd Tadalafil 20 mg qd Combination	18 19 21	12 weeks	IPSS, Qmax, PVR, QoL, IIEF, AEs	3
Ng	2009	China	50–80	BPH	/	Yes	/	RCCT	Doxazosin0.4–0.8 mg qd + vardenafil 10 mg qd	37	2 days	AEs	6
									Doxazosin0.4–0.8 mg qd + placebo	37			
Regadas	2013	Brazil	> 45	BPH	> 14	/	≥6 months	RCT	Tamsulosin 0.4 mg qd + tadalafil 5 mg qd	20	30 days	IPSS, Qmax, QoL, AEs	5
									Tamsulosin 0.4 mg qd	20			
Gacci	2012	Italy	40–80	BPH	≥12	Yes/No	/	RCT	Vardenafil 10 mg qd + tamsulosin 0.4 mg qd	30	2 weeks	IPSS, Qmax, PVR, QoL, IIEF, AEs	5
									Tamsulosin 0.4 mg qd + Placebo	30			
Kumar	2014	India	> 50	BPH	≥8	/	/	RCT	Alfuzosin 10 mg qd	25	12 weeks	IPSS, IIEF, Qmax, PVR, QoL	5
									Tadalafil 10 mg qd	25			
									Combination	25			
Jin	2011	China	50–75	BPH	≥10	Yes	/	MRCT	Doxazosin 4 mg qd + sildenafil 25–100 mg on demand	168	6 months	IPSS, QoL, IIEF, AEs	3
									Sildenafil 25–100 mg on demand	82			

*LUTS* lower urinary tract symptom, *BPH* benign prostatic hyperplasia, *ED* erectile dysfunction, *Qmax* maximum flow rate, *IPSS* international prostase Symptom score, *PVR* postvoid residual urine, *QoL* quality of life, *IIEF* international index of erectile function, */* not available, *AEs* adverse events, *RCT* randomized controlled trial, *MRCT* multicenter randomized controlled trial, *RCCT* randomized controlled crossover trial

### Quantitative synthesis

#### PDE5-is versus ABs for lower ureteric stones

**Fig. 2:**
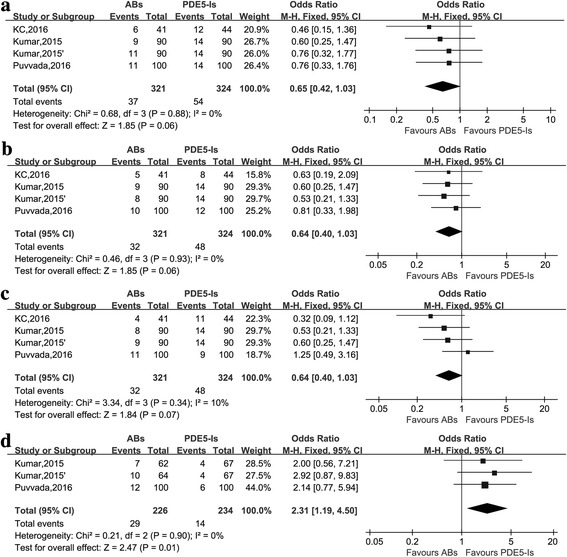
The comparisons between PDE5-Is and ABs for treating lower ureteric stones **a** Headache **b** Dizziness **c** Backache **d** Abnormal ejaculation Kumar, 2015: tamsulosin vs tadalafil. Kumar, 2015′: silodosin vs tadalafil

As displayed in Fig. 2 and Additional file 1: Table S1, baseline characteristics, treatment outcomes and AEs were not statistically different except for the abnormal ejaculation between the two groups. There was a trend that ABs had a lower incidence of headache, dizziness and backache. Combining the results of included studies, PDE5-Is was comparable on the efficacy of lower ureter stones passage and had a significantly lower rate of abnormal ejaculation (2.31[1.19 to 4.50]; *P* = 0.01) than ABs. Sensitivity analysis was conducted by excluding each of the 4 studies.When excluding the study of Kumar (2015′) et al. [13] the pooled odds ratio (OR) of expulsion rate and expulsion time was 0.37 (95%CI: 0.21–0.65, *P* = 0.0005) and 1.90 (95%CI: 0.98–2.82, *P* < 0.0001), respectively, demonstrating tadalafil might have better expulsion effect and shorter expulsion time than tamsulosin.

#### Tadalafil plus tamsulosin versus tamsulosin for lower ureteric stones

**Fig. 3:**
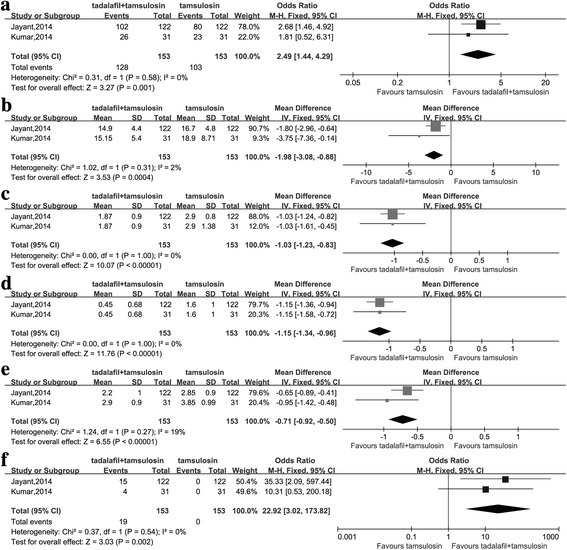
The comparisons between combination therapy and tamsulosin monotherapy for treating lower ureteric stones **a** Expulsion rate **b** Expulsion time **c** Analgesic use **d** No. of colic episodes **e** No. of hospital visits **f** Improvement in ED

As displayed in Fig. 3 and Additional file 1: Table S1, the pooled WMD for expulsion time, no. of hospital visits, no. of colic episodes and analgesic use was − 1.98 (95%CI: -3.08--0.88, *P* = 0.0004), − 0.71 (95%CI: -0.92--0.50, *P* < 0.0001), − 1.15 (95%CI: -1.34--0.96, *P* < 0.0001) and − 1.03 (95%CI: -1.23--0.83, P < 0.0001), respectively and the pooled OR for expulsion rate and Improvement in ED was 2,49 (95%CI: 1.44–4.29, *P* = 0.001) and 22.92 (95%CI: 3.02–173.82, *P* = 0.002), respectively. The resultsindicated the combination therapy of tadalafil and tamsulosin was more effective on treating ED and lower ureteric stones without higher rate of AEs than tamsulosin. Sensitivity analysis was not performed because only 2 studies were included.

#### PDE5-is versus ABs for LUTS/BPH

**Fig. 4:**
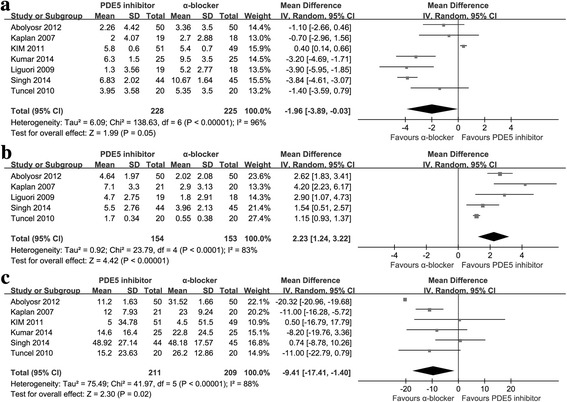
The comparisons between PDE5-Is and ABs for treating LUTS/BPH **a** Change of IPSS **b** Change of IIEF score **c** Change of PVR

As displayed in Fig. 4 and Additional file 1: Table S1, ABs was significantly more effective than PDE5-Is on decreasing PVR (− 9.41 [− 17.41 to − 1.40]; *P* = 0.02) and IPSS (− 1.96 [− 3.89 to − 0.03]; *P* = 0.05), while PDE5-Is showed greater effect than ABs on increasing IIEF score (2.23 [1.24 to 3.22]; *P* < 0.0001). Sensitivity analysis was conducted, and when ruling out the study of Kim et al. [29] the pooled WMD of change of Qmax was − 0.78 (95%CI:-1.41--0.15,P = 0.02) which meant ABs might have a better effect in increasing Qmax. The pooled WMD of the change of PVR was − 8.58 (95%CI: -19.34-2.19, *P* = 0.12) after excluding the low-quality study of Kaplan et al. [32] which indicated there might have no statistical significance on decreasing PVR. When ruling out the three low-quality studies of Kaplan et al. [32],Singh et al. [31] and Liguori et al. [36] the pooled WMD of change of IPSS was − 1.24 (95%CI:-3.11--0.79,*P* = 0.19) which meant ABs might not have a better effect in decreasing IPSS.

#### PDE5-is plus ABs versus ABs for LUTS/BPH

**Fig. 5:**
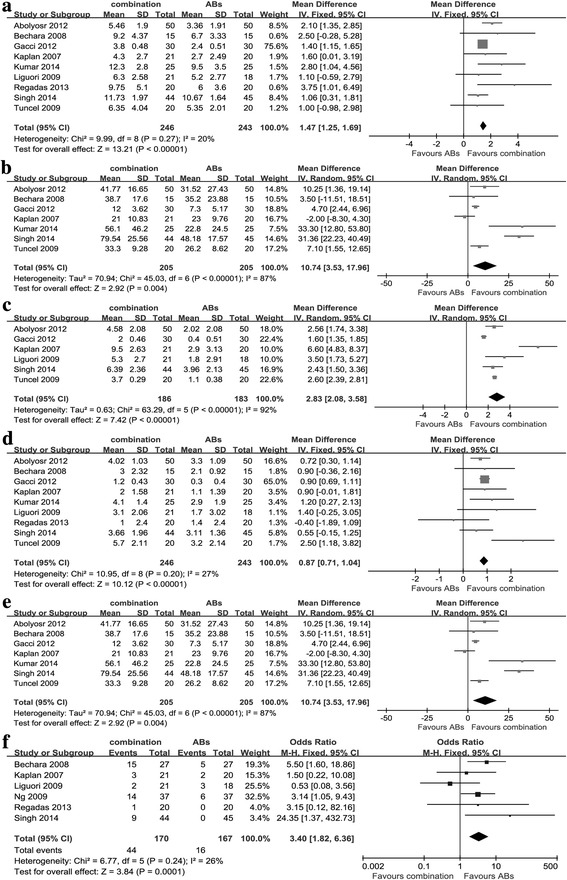
The comparisons between combination therapy and ABs for treating LUTS/BPH **a** Change of IPSS **b** Change of QoL **c** Change of IIEF score **d** Change of Qmax **e** Change of PVR. **f** AEs

As displayed in Fig. 5 and Additional file 1: Table S1, the pooled WMD for change of IPSS, QoL, IIEF score, Qmax and PVR was 1.47 (95%CI: 1.25–1.69, *P* < 0.0001), 0.59 (95%CI: 0.22–0.97, *P* = 0.002), 2.83 (95%CI: 2.08–3.58, *P* < 0.0001), 0.87 (95%CI: 0.71–1.04, *P* < 0.0001) and 10.74 (95%CI: 3.53–17.96, *P* = 0.004), respectively, indicating PDE5-Is plus ABs had better effect on improving LUTS/BPH than ABs alone. PDE5-Is plus ABs had higher incidences of AEs (3.69 [2.38 to 5.74]; *P* < 0.0001), headache (4.87 [2.28 to 5.74]; *P* < 0.0001) and dyspepsia (6.67 [1.46 to 30.55]; *P* = 0.01) than ABs alone. Sensitivity analysis was conducted and there was no significant change.

#### PDE5-Is plus ABs versusPDE5-Is for LUTS/BPH

**Fig. 6:**
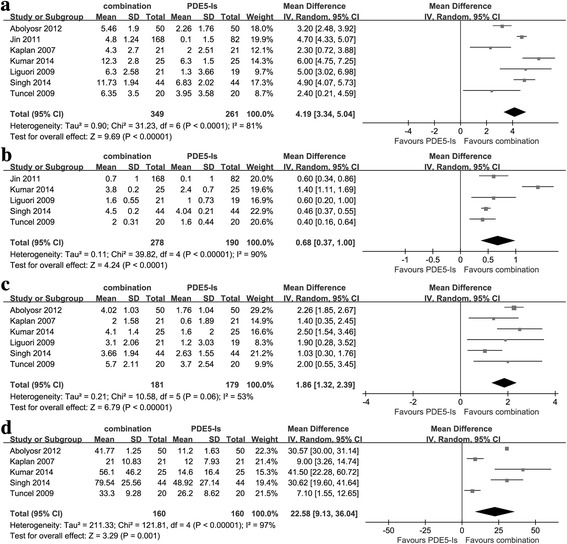
The comparisons between combination therapy and PDE5-Is for treating LUTS/BPH **a** Change of IPSS **b** Change of QoL **c** Change of Qmax **d** Change of PVR

As displayed in Fig. 6 and Additional file 1: Table S1, the pooled WMD for change of IPSS, QoL, Qmax and PVR was 4.19 (95%CI: 3.34–5.04, P < 0.0001), 0.68 (95%CI: 0.37–1.00, P < 0.0001), 1.86 (95%CI: 1.32–2.39, *P* < 0.0001) and 22.58 (95%CI: 9.13–36.04, *P* = 0.001), respectively, indicating PDE5-Is plus ABs had a significantly better effect on improving LUTS/BPH and ED without increased incidences of AEs than PDE5-Is alone. Sensitivity analysis was conducted and there was no significant change.

## Discussion

This is the first systematic review and meta-analysiscomparing the efficacy and safety of monotherapy and combination therapy of PDE5-Is and ABs for treating lower ureteric stones. Meanwhile, it is an update in LUTS/BPH. A few meta-analyses had analyzed the effect of PDE5-Is for treating LUTS/BPH when compared with ABs or placebo [42–44]. In 2015, XH Wang et al. worked out several different conclusions comparing with our analysis in LUTS/BPH which could mainly attribute to the incomplete literature search. However, we made an integrated and high-quality literature search and most of the included studies were high-quality RCTs.

As for LUTS/BPH, our pooled results suggested that ABs had a significantly better effect than PDE5-Is on the reduction of IPSS and PVR without significant difference of Qmax and QoL. Meanwhile, PDE5-Is had a statistically significant better effect than ABs on improving IIEF score. This suggested PDE5-Is could exert different therapeutic effect by relieving the obstruction of prostate and relaxing the bladder SMs [45]. The relaxation of PDE5-Is in the detrusor muscle could withstand the relaxation of prostate and bladder neck, which lessen the effect of urodynamics, especially PVR and IPSS [46].We also found that combination therapy had the best effect on reducing IPSS, PVR, and on increasing Qmax and QoL compared with either of monotherapy, while the combination therapy was significantly more effective on improving IIEF score compared with ABs. The results might demonstrate the therapy of tadalafil daily don’t have a negative impact on bladder contractility and outlet condition [47]. No significant difference on increasing IIEF score between combination therapy and monotherapy of PDE5-Is was found, which demonstrated that ABs had little power to improve ED. Significant heterogeneity was detected among treatment outcomes of all the three comparisons for treating LUTS/BPH, which might due to various methods and doses, quality, and duration of intervention.

It is important that our meta-analysis revealed that the combination therapy for treating LUTS/BPH showed better effect than either monotherapy on reducing IPSS and PVR and increasing QoL and Qmax. The best effect may result from the synergistic function of NO-mediated relaxation effect by PDE5-Is, and reduction of the sympathetic tone mediated by α-adrenergic receptors blocking by ABs of the identical SMs in the bladder neck and prostate [32]. Also, our results corroborated the efficacy of combination therapy for patients suffered from LUTS and ED. In the comparison of PDE5-Is with ABs for LUTS/BPH, we found that ABs had significantly better effect on increasing Qmax than PDE5-Is after removing the study of Kim et al. and the pooled WMD for change of PVR turned to be non-significant after removing the study of Kaplan et al., which could be explained by large sample size and low quality, respectively. When ruling out all the three low-quality studies of Kaplan et al. [32], Singh et al. [31] and Liguori et al. [36], the pooled WMD of change of IPSS turned to be non-significant, which meant ABs might not have a better effect in decreasing IPSS.

Variations of the outcomes could also be explained by different baseline characteristics such as age, BMI, and initial risk factors for LUTS/BPH.

It is clear that substantial work has been conducted to verify the relationship between ED and LUTS/BPH. Both of them are highly prevailing in older men and are closely linked [48], independently of cardiovascular problems, as confirmed by numerous epidemiology researches [10, 49–51]. At present, the connection between ED and LUTS/BPH is supported by four primary mechanisms in the penis and prostate as followed: ①the rhokinase activation/endothelin pathway; ②the metabolic syndrome hypothesis and autonomic hyperactivity; ③the physiopathologic consequences of pelvic atherosclerosis; ④changes in the NO/cGMP pathway [52]. PDE5 isoenzymes play a role in the metabolisms and exist in the human bladder, prostate and urethra. Moreover, there is increasing evidence that PDE5-Is may be effective for LUTS/BPH [53–55].

As for AEs, our pooled results suggested that PDE5-Is plus ABs had a higher incidence of total AEs, headache and dyspepsia than ABs without significant difference in other two comparisons of LUTS/BPH, which suggested the addition of PDE5-Is to ABs could increase risks of the AEs in treating LUTS to some extent. It is wise to be prudent in comparing the AEs of similar medicines even though tiny differences in the mechanisms. In the study of XH Wang et al., the occurrence rate of AEs for the combination treatment was only numerically greater than either of monotherapy, and this might due to the inadequate size of the sample. Based on the present RCTs, most related AEs of cases were slight or moderate and only a few patients discontinued due to AEs. Therefore, the overall safety profile of the two classes of drugs was good.

As for lower ureteric stones, the pooled results of this study indicated that tadalafil had better expulsion effect and shorter expulsion time than tamsulosin, and tadalafil had a lower incidence of abnormal ejaculation compared with ABs, which could be explained that tadalafil played a better role in relaxing SMs of posterior urethra than that of anterior urethra and bladder neck. This result reminds us that prescribing ABs for patients with lower ureteric stones and ejaculatory dysfunction should be prudent, and tadalafil may be a good substitute. And tadalafil plus ABs had significantly better improvement on IIEF score, higher expulsion rate, shorter expulsion time, less analgesic use and fewer hospital visits than ABs monotherapy without increased AEs. Our results demonstrated that tadalafil had an impressive improvement on expelling lower ureteric stones when combined with ABs and the combination therapy was safe for patients. Significant heterogeneity was observed among treatment outcomes of comparing ABs with PDE5-Is, which might due to various methods and duration of intervention.

At present, two convincing mechanisms of tadalafil in expelling lower ureteric stones are as followed: (1) slight-to-moderate relaxation of SMs; (2) extension of local blood vessels which increases blood perfusion [56–58]. Increased NO/cGMP concentration could relax the SMs in the prostate, urethra and bladder,and increased blood perfusion may relieve intraprostatic inflammation, ureter spasms and mucosal edema, which may contribute to the expulsion of lower ureteric stones.

Unfortunately, the important issue of PDE5-Is, the daily cost, has not been investigated, and none of the included RCTs had a performed cost analysis. The cost of drug therapy is directly related to the long-term efficacy and safety profile, and the QoL of men treated with PDE5-Is alone or in combination with other drugs in continuous administration.

Nevertheless, there were several main limitations when analyzing and interpreting results in our present systematic review and meta-analysis. The major limitation was the quantity and various qualities and it was difficult to perform subgroup analysis to evaluate the heterogeneity among the included studies. In Table 1, we evaluated the quality by Jadad score and 4 RCTs had got 3 points which meant low-quality, which may limit the quality grade of evidence although other studies were evaluated as high-quality. Secondly, short duration and small populations also had a huge impact on the overall results. Thirdly, uncontrollable lifestyle modifications might influence the results. In our study, there were significant changes when removing some certain studies, which indicated there existed instability in our consequences, which might due to small sample size, inconsistent quality and the heterogeneity of the included original RCTs.

In the further, well-designed, prospective, multicenter randomized control studies with data of cost analysis, longer duration and larger sample size, and fundamental researches surveying mechanisms of PDE5-Is treating LUTS/BPH and lower ureteric stones, are required to help us better demonstrate the advantages as well as drawbacks of combination drug therapies. Clinical trials on the basis of the highest quality standard and method should be encouraged to ensure that the results have statistical significance and clinical relevance at the same time.

## Conclusion

In conclusion, our study indicated that combination therapy of PDE5-Is and ABs had the best effect on improving LUTS/BPH or expulsing lower ureteric stones. As for monotherapy therapy, ABs had a better effect on improving LUTS/BPH and PDE5-Is were more effective on treating ED. Monotherapy use of PDE5-Is was effective on improving LUTS/BPH except for less reduction of PVR and IPSS as compared with ABs. Monotherapy of tadalafil had a better effect on expulsing lower ureteric stones than tamsulosin and had a lower incidence of abnormal ejaculation than ABs. What’s more, monotherapy or combination therapy was safe and tolerant. Our results affirmed the therapeutic effect and safety of PDE5-Is and ABs, and provided evidences for drug treatment and update of guideline of LUTS/BPH or lower ureteric stones.

## Additional file

Additional file 1: **Table S1.** Outcomes including baseline characteristics, treatment outcomes and adverse effects of this study. *ABs* α-1 blockers, *PDE5-Is* phosphodiesterase 5 inhibitors, *BPH* benign prostatic hyperplasia, *LUTS* lower urinary tract symptoms, *ED* erectile dysfunction, *OR* odds ratio, *WMD* weighted mean difference, *CI* confidence interval, *IPSS* International Prostate Symptom Score, *PVR* postvoid residual urine, *Qmax* maximum flow rate, *IIEF* International Index of Erectile Function, *QoL* quality of life, *AE*s adverse effects. (DOCX 27 kb)

## Abbreviations

ABs: Adrenoceptor1 blockers
AEs: Adverse events
BPH: Benign prostatic hyperplasia
cGMP: Cyclic-guanine monophosphate
CI: Confidence interval
EAU: European association of urology
ED: Erectile dysfunction
IIEF: International index of erectile function
IPSS: International prostate symptom score
JUA: Japanese urological association
LUTS: Lower urinary tract symptoms
NO: Nitric oxide
OR: Odds ratio
PDE5-Is: phosphodiesterase5 inhibitors
PVR: Post-void residual
Qmax: Maximum flow rate
QOL: Quality of life
RCT: Randomized controlled trials
SM: Smooth muscle
WMD: Weighted mean difference
