# Supplementary material for: Efficacy and safety of PDE5-Is and α-1 blockers for treating lower ureteric stones or LUTS: a meta-analysis of RCTs
Source: BMC Urol. 2018 May 3;18:30. doi: 10.1186/s12894-018-0345-4 (PMC5934901; doi:10.1186/s12894-018-0345-4)
Supplement: Supplementary file 1 — Table S1. Outcomes including baseline characteristics, treatment outcomes and adverse effects of this study. ABs α-1 blockers, PDE5-Is phosphodiesterase 5 inhibitors, BPH benign prostatic hyperplasia, LUTS lower urinary tract symptoms, ED erectile dysfunction, OR odds ratio, WMD weighted mean difference, CI confidence interval, IPSS International Prostate Symptom Score, PVR postvoid residual urine, Qmax maximum flow rate, IIEF International Index of Erectile Function, QoL quality of life, AEs adverse effects. (DOCX 27 kb) [file 12894_2018_345_MOESM1_ESM.docx]

Table S1. Outcomes including baseline characteristics,treatment outcomes and adverse effects of this study.

| **Outcome** | | | **NO. of  studies** | **NO. of  patients** | **OR/WMD (95% CI)** | **p-value** | **Study heterogeneity** | |
| --- | --- | --- | --- | --- | --- | --- | --- | --- |
|  |  |  |  |  |  |  | *I*2 | p-value |
| **ABs  VS  PDE5-Is for treating stones** | **Baseline characteristics** | Age(years) | 4 | 321/324 | -0.19(-2.09,1.71) | 0.85 | 0% | 0.79 |
|  |  | Proportion of males | 4 | 321/324 | 0.98(0.70,1.37) | 0.92 | 0% | 0.54 |
|  |  | Stone size (mm3) | 4 | 321/324 | -0.14(-0.35,0.06) | 0.17 | 12% | 0.33 |
|  | **Treatment outcomes** | Expulsion rate | 4 | 321/324 | 0.75(0.31,1.82) | 0.52 | 83% | 0.0005 |
|  |  | Expulsion time (day) | 4 | 321/324 | 0.62(-1.03,2.26) | 0.46 | 86% | 0.0001 |
|  |  | Analgesic use (mg) | 4 | 321/324 | -4.78(-16.20,6.65) | 0.41 | 98% | <0.0001 |
|  |  | Episodes of  colic pain | 4 | 321/324 | 0.08(-0.72,0.88) | 0.85 | 97% | <0.0001 |
|  | **Adverse effects** | Headache | 4 | 321/324 | 0.65(0.42,1.03) | 0.06 | 0% | 0.88 |
|  |  | Dizziness | 4 | 321/324 | 0.64(0.40,1.03) | 0.06 | 0% | 0.93 |
|  |  | Orthostatic hypotension | 4 | 321/324 | 0.68(0.39,1.19) | 0.18 | 0% | 0.39 |
|  |  | Backache | 4 | 321/324 | 0.64(0.40,1.03) | 0.07 | 10% | 0.34 |
|  |  | Abnormal ejaculation | 3 | 226/234 | 2.31(1.19,4.50) | 0.01 | 0% | 0.9 |
| **tamsulosin and tadalafil VS tamsulosin for treating stones** | **Baseline characteristics** | Age(years) | 2 | 153/153 | 1.18(-1.14,3.76) | 0.37 | 0% | 0.54 |
|  |  | Proportion of males | 2 | 153/153 | 1.24(0.79,1.96) | 0.35 | 50% | 0.16 |
|  |  | Stone size (mm3) | 2 | 153/153 | 0.19(-0.16,0.53) | 0.29 | 62% | 0.1 |
|  | **Treatment outcomes** | Expulsion rate | 2 | 153/153 | 2.49(1.44,4.29) | 0.001 | 0% | 0.58 |
|  |  | Expulsion time (days) | 2 | 153/153 | -1.98(-3.08,-0.88) | 0.0004 | 2% | 0.31 |
|  |  | Analgesic use (mg) | 2 | 153/153 | -1.03(-1.23,-0.83) | <0.0001 | 0% | 1 |
|  |  | No. of colic episodes | 2 | 153/153 | -1.15(-1.34,-0.96) | <0.0001 | 0% | 1 |
|  |  | No. of hospital visits | 2 | 153/153 | -0.71(-0.92,-0.50) | <0.0001 | 19% | 0.27 |
|  |  | Improvement in ED | 2 | 153/153 | 22.92(3.02,173.82) | 0.002 | 0% | 0.54 |
|  | **Adverse effects** | Headache | 2 | 153/153 | 1.13(0.69,2.51) | 0.41 | 0% | 0.99 |
|  |  | Dizziness | 2 | 153/153 | 1.13(0.69,2.51) | 0.41 | 0% | 0.99 |
|  |  | Orthostatic  hypotension | 2 | 153/153 | 1.85(0.61,5.65) | 0.28 | 0% | 0.92 |
|  |  | Backache | 2 | 153/153 | 1.71(0.86,3.41) | 0.13 | 0% | 0.95 |
|  |  | Abnormal ejaculation | 2 | 153/153 | 0.63(0.34,1.19) | 0.16 | 0% | 0.97 |
| **PDE5-Is  VS  ABs  for treating LUTS/BPH** | **Baseline characteristics** | Age(years) | 5 | 155/152 | 1.08(-0.45,2.61) | 0.17 | 26% | 0.25 |
|  |  | Age<60y | 3 | 84/83 | 0.94(0.51,1.72) | 0.83 | 0% | 0.58 |
|  |  | With hypertension | 3 | 84/83 | 0.74(0.38,1.44) | 0.37 | 0% | 0.82 |
|  |  | With diabetes mellitus | 3 | 84/83 | 1.27(0.79,2.04) | 0.87 | 0% | 0.98 |
|  |  | Total IPSS | 5 | 185/182 | -0.10(-1.12,0.92) | 0.85 | 9% | 0.35 |
|  |  | QoL | 3 | 83/83 | 0.16(-0.01,0.33) | 0.06 | 0% | 0.97 |
|  |  | PVR | 5 | 186/184 | -2.11(-10.76,6.53) | 0.63 | 65% | 0.02 |
|  |  | Qmax | 6 | 205/202 | -0.32(-0.98,0.33) | 0.34 | 21% | 0.28 |
|  |  | IIEF score | 5 | 154/153 | -0.08(-0.39,0.24) | 0.63 | 40% | 0.16 |
|  | **Treatment outcomes** | Change of total IPSS | 7 | 228/225 | -1.96(-3.89,-0.03) | 0.05 | 96% | <0.0001 |
|  |  | Change of QoL | 5 | 159/157 | 0.02(-0.28,0.32) | 0.87 | 95% | <0.0001 |
|  |  | Change of IIEF score | 5 | 154/153 | 2.23(1.24,3.22) | <0.0001 | 83% | <0.0001 |
|  |  | Change of Qmax | 7 | 228/225 | -0.51(-1.34,0.32) | 0.23 | 91% | <0.0001 |
|  |  | Change of PVR | 6 | 211/209 | -9.41(-17.41,-1.40) | 0.02 | 88% | <0.0001 |
|  | **Adverse effects** | Total | 2 | 72/69 | 0.55(0.24,1.30) | 0.17 | 0% | 0.42 |
|  |  | Headache | 2 | 72/69 | 4.05(0.44,37.31) | 0.22 | 0% | 0.8 |
| **ABs and PDE5-Is  VS  ABs  for treating LUTS/BPH** | **Baseline characteristics** | Age(years) | 5 | 135/133 | 0.23(-1.37,1.84) | 0.78 | 13% | 0.33 |
|  |  | Total IPSS | 5 | 156/153 | 0.59(-0.49,1.68) | 0.28 | 0% | 0.92 |
|  |  | QoL | 3 | 85/83 | 0.11(-0.06,0.29) | 0.2 | 25% | 0.26 |
|  |  | PVR | 5 | 165/165 | 4.63(-5.28,14.54) | 0.36 | 91% | <0.0001 |
|  |  | Qmax | 7 | 206/203 | -0.25(-0.95,0.45) | 0.48 | 55% | 0.04 |
|  |  | IIEF | 6 | 186/183 | 0.19(-0.67,1.04) | 0.67 | 74% | 0.002 |
|  | **Treatment outcomes** | Change of total IPSS | 9 | 246/243 | 1.47(1.25,1.69) | <0.0001 | 20% | 0.27 |
|  |  | Change of QoL | 7 | 175/173 | 0.59(0.22,0.97) | 0.002 | 90% | <0.0001 |
|  |  | Change of IIEF score | 6 | 186/183 | 2.83(2.08,3.58) | <0.0001 | 92% | <0.0001 |
|  |  | Change of Qmax | 9 | 246/243 | 0.87(0.71,1.04) | <0.0001 | 27% | 0.2 |
|  |  | Change of PVR | 7 | 205/205 | 10.74(3.53,17.96) | 0.004 | 87% | <0.0001 |
|  | **Adverse effects** | Total | 6 | 170/167 | 3.40(1.82,6.36) | 0.0001 | 26% | 0.24 |
|  |  | Headache | 4 | 128/129 | 10.64(2.52,44.97) | 0.001 | 8% | 0.34 |
|  |  | Dizziness | 4 | 93/82 | 0.66(0.19,2.35) | 0.52 | 7% | 0.34 |
|  |  | Hypotension | 4 | 106/97 | 1.28(0.18,9.36) | 0.81 | 0% | 0.45 |
|  |  | Dyspepsia | 3 | 92/92 | 6.67(1.46,30.55) | 0.01 | 0% | 0.7 |
| **ABs and PDE5-Is  VS  PDE5-Is   for treating LUTS/BPH** | **Baseline characteristics** | Age(years) | 4 | 254/166 | 0.26(-0.96,1.49) | 0.67 | 0% | 0.43 |
|  |  | Total IPSS | 5 | 304/216 | 1.03(0.13,1.92) | 0.02 | 0% | 0.61 |
|  |  | QoL | 3 | 85/83 | -0.08(-0.26,0.10) | 0.4 | 0% | 0.55 |
|  |  | PVR | 5 | 303/217 | 3.35(2.08,5.02) | <0.0001 | 0% | 0.41 |
|  |  | Qmax | 6 | 324/236 | -0.24(-0.60,0.13) | 0.2 | 54% | 0.05 |
|  |  | IIEF score | 5 | 156/154 | -0.10(-0.42,0.21) | 0.53 | 0% | 0.44 |
|  | **Treatment outcomes** | Change of total IPSS | 7 | 349/261 | 4.19(3.34,5.04) | <0.0001 | 81% | <0.0001 |
|  |  | Change of QoL | 5 | 278/190 | 0.68(0.37,1.00) | <0.0001 | 90% | <0.0001 |
|  |  | Change of IIEF score | 5 | 156/134 | 0.47(-0.10,1.05) | 0.11 | 46% | 0.13 |
|  |  | Change of Qmax | 6 | 181/179 | 1.86(1.32,2.39) | <0.0001 | 53% | 0.06 |
|  |  | Change of PVR | 5 | 160/160 | 22.58(9.13,36.04) | 0.001 | 97% | <0.0001 |
|  | **Adverse effects** | AEs | 3 | 86/84 | 1.19(0.51,2.78) | 0.69 | 0% | 0.85 |
|  |  | Dyspepsia | 3 | 233/147 | 1.52(0.58,3.98) | 0.4 | 0% | 0.94 |

***ABs*** α-1 blockers, ***PDE5-Is*** phosphodiesterase 5 inhibitors, ***BPH*** benign prostatic hyperplasia, ***LUTS*** lower urinary tract symptoms, ***ED*** erectile dysfunction, ***OR*** odds ratio, ***WMD*** weighted mean difference, ***CI*** confidence interval, ***IPSS*** International Prostate Symptom Score, ***PVR*** postvoid residual urine, ***Qmax*** maximum flow rate, ***IIEF*** International Index of Erectile Function, ***QoL*** quality of life, ***AE***s adverse effects
